# Observations on Some Epidemical Effects

**Published:** 1792

**Authors:** William Blizard

**Affiliations:** Corresponding Member of the Royal Society of Sciences of Gottingen, and Surgeon to the London Hospital.


X. *Observations on some epidemical Effects. By Mr.* William Blizard, *F. R. S. and S. A. Corresponding Member of the Royal Society of Sciences of Gottingen, and Surgeon to the London Hospital.*

MANKIND are generally interested in inquiries into the nature of epidemical effects upon the human body: it is, therefore, to be lamented that the labourers have been so few in this field of medical science.

If nature were strictly watched, there might possibly in time be ascertained some connexion and order in epidemical causes and effects; but from the obscurity of many of the operations, in maintaining the various balances among the parts of matter, much industry and attention are necessary in this undertaking. The observations of many men, in various parts, will be required, and great philosophical acumen for arranging and comparing the facts and drawing general general inferences. The laws of the animal œconomy, and particularly thofe that direct the conftant efflux from exhalent arteries, and the continual influx into the abforbents, fo expreffive of the connexion of the body with furrounding matter, muft be underftood. The weight and temperature of the air, its moifture, and what elfe it may contain, muft be continually fubjects of inveftigation. The winds too, as affecting the properties of the air, and directing the courfe of matter floating in it, muft not be lefs regarded: but as all the parts of nature are linked together, are dependent upon, and in their changes affect each other, the powers of the mineral and vegetable kingdoms upon the air and body muft alfo be confidered; nor fhould the influence of the planetary fyftem be unheeded: for, doubtlefs, effects as certain, though lefs manifeft than the feafons and their varieties, arife from the courfes of orbs, whofe diftances, or lengths of periods of revolution, make them but little the objects of philofophical attention*.

Accurate

* The meteorological journals publifhed annually by the Royal Society, and other philofophical bodies, will, in thefe views, probably, at fome period, be experienced of invaluable

Accurate inquiries into the nature of endemic diſeaſes, and their gradations of diminution and change from country to country, may alſo throw great light on the nature and power of the many agents in the production of epidemical affections.

Nor ſhould the inquirer be diſcouraged from any conſiderations of difficulty or improbability of ultimate complete ſucceſs; for mankind could not fail of being benefited by more accurate and connected hiſtories of epidemical effects, though the nature of their cauſes ſhould never be unveiled.

In this uncultivated field of inquiry, then, any obſervations accurately made and faithfully recorded, cannot be altogether uſeleſs: and trials of application of the laws of the animal œconomy, in accounting for epidemical effects, may alſo perhaps, at ſome period, be uſeful, either in confirming truth or in expoſing error.

On theſe preſumptions the following ſentiluable utility. The Marine Society, deſirous of being ſerviceable to the world in every way compatible with their great deſign, have alſo reſolved on having an accurate meteorological record kept on board their ſhip moored between Deptford and Greenwich.

ments are ventured. The fubject being involv-ed in much obfcurity, attempts to throw light upon it, though without fuccefs, may furely be pardoned.

" *Quale per incertam lunam fub luce maligna*

" *Eft iter in fylvis.*"

However far the vafcular fyftem be divided, each part may be confidered as having a function affigned to its performance different from that of every other portion. Every line, therefore, of an artery has a degree and kind of irritability by which it is regularly obedient to the quantity and nature of the fluid that paffes through it. The fame may alfo, perhaps, be obferved of the veins and abforbents; for fhould proper mufcular coats to thefe veffels be not allowed, yet caufes affecting the arteries that fupply their membranous fides, or that are fituated near them, muft produce fome change in the offices of the tubes themfelves.

The many kinds and degrees of abftractions made from the blood, in its courfe through the arteries, and the various additions received in its progrefs in the veins, muft conftantly be productive of alterations in that fluid; fo that in no two parts into which any veffel can be divided is the fluid exactly the fame: and thus the blood, in all the feries of veffels and ftructures of parts, is determined precifely as a proper exciter to the degree and kind of irritability of the part to be excited.

The peculiar effects of ftimulants arife then from the peculiar difpofitions of parts of the vafcular fyftem; fo that the veffels of one organ may be powerfully excited, while every other part, equally fubjected to the influence of the exciting caufe, fhall remain unaffected. Effects of this nature are generally underftood, and thence claffifications of the materia medica. The effect diftinguifhed by the term fedative, is undoubtedly as certain and as relative to the ftates of different parts, as the effects of other defcriptions of ftimulants *.

Thus the harmony of the animal machine depends on a precife relation of the irritability of the infinite diftinctions of parts; and on the

* Obfervations feem to prove that uva urfi, *e. g.* diminifhes or alters the irritable difpofition of the vefical veffels. Cantharides peculiarly affect thefe parts, producing contraction and pain. Will it, then, appear extraordinary if a caufe, or chain of caufes of a general nature fhould eventually fo difpofe thefe parts, as that the flighteft incidental matter fhould excite inflammation and its train of fymptoms?

condition, condition, quantity, and motion of the blood as exactly suitable to that irritability.

An epidemical caufe, therefore, that alters the irritability of the veffels; that changes the nature, quantity, or motion of the blood, as relative to them; or that directly and unufually acts upon them, muft produce a change in the difpofition or function of an organ fo affected.

It appears that epidemical caufes fometimes act upon parts of the vafcular fyftem, fo as to render them in an extraordinary degree fufceptible of impreffions from occafional caufes*. And that when an occafional caufe happens, the effects are determined, and characterifed, by the organization, ftate, and function of the affected part †.

It has long been obferved, that difeafes of every kind partake of the nature of the prevailing

* The general irritable difpofition of particular parts is oftentimes ftrongly expreffed in gouty inflammation of the extremities, rheumatic inflammation of the large joints, whitlow, boil, inflamed eyes, piles, various complaints of the fkin, &c.: for the fligheft accidental caufes fhall in fome feafons occafion one or other of thefe complaints very generally.

† The conditions of ulcer, fcirrhus, cancer, &c., and the progrefs and events of cafes of fracture, wound, &c., experience proves depend in fome degree on caufes of general influence not underftood.

epidemic; epidemic: but when there is no manifestation of a reigning epidemic, obscure causes, not equal to the production of a defined disease, appear frequently to affect constantly occurring disorders, so as greatly to diversify their symptoms, and determine their events*.

When occasional causes, and those of weak power, affect bodies of any kind of hereditary disposition, at the same time, *generally*, which *individually* have seldom at any other period been affected by similar causes, there must have been a previous change produced by agents of general influence; and thus hereditary dispositions may be increased, and also diminished, or totally changed through the influence of epidemical causes†.

* *e. g.* The general violence of the small pox, from inoculation, is very different at times. The disposition of bodies to produce the disease is also observed to be in various degrees; there being sometimes such repugnance to the infection, that the insertion of the virus shall repeatedly fail in its effects. When the disease is brought on, under such circumstances, it is remarked to be proportionally mild in its symptoms.

† This is illustrated in scrophulous habits; of which many are remarked to present themselves at the hospital, within short spaces of time, principally on account of the same pressing symptoms. The more lasting changes in such habits are of common observance.

The

The following facts ſuggeſted the foregoing reflections:

In the autumn of 1787 a man was admitted into the London Hoſpital on account of a hurt of the head. After a few days a conſiderable degree of eryſipelatous inflammation appeared over the whole ſcalp. The evacuations were larger than would have been judged proper had the nature of the ſymptom been clearly underſtood. The man died. Eryſipelas ſoon generally appeared both in the hoſpital and out of it; and almoſt every caſe of injury of the ſcalp, however ſlight, was attended with more or leſs inflammation of the eryſipelatous kind *.

In the beginning of the year 1789, in ſeveral recent caſes of ſyphilis, in which mercury was in uſe, diſtreſſing ulcerations originated in the tonſils, which became worſe during the employment of the mineral. On diſcontinuing the mercurial preparations, employing the warm

* Contemporary inſtances of gouty inflammation, frequently obſervable in conſequence of ſlight caſual irritation, ſeem alſo ſtrikingly to evince the power of epidemical cauſes in prediſpoſing parts to be variouſly affected.

bath, bath, and washing with soft gargarisms, they all got well *.

The disease called the Mumps *(Cynanche parotidea)* is understood to be frequently succeeded by symptoms of inflammation in the breasts of women and *testes* of men. A few years since this complaint appeared in several patients at the hospital. Not one of these cases was succeeded by the different symptoms in the sexes; but at the very same time there was a remarkable number of instances of inflamed breasts and testes without any known cause whatever †.

Hernia humoralis, and spasmodic affections about the neck of the bladder and urethra are sometimes of general occurrence. In January, 1791, they were common symptoms of gonorrhœa. About the same time also cases of suppression of urine frequently happened without the least suspicion of gonorrhœal taint.

* These ulcerations were widely different from those about the mouth which are of common occurrence in mercurial courses.

† Instances of this kind, and of the retrocession of gouty and erythematous inflammation, may possibly one day prove some kind of similarity of structure or function, in distant parts, affected together, or in succession, by the same causes.

n two cafes of fimple fracture of the thigh, at the fame time in the hofpital, without comminution or much contufion, delirium took place within a few days of the accidents. The like fymptom occurred at another hofpital, at the fame period, in feveral cafes of fimple fracture. In thefe inftances the delirium fubfided, and the patients did well, without any other remarkable event.

A difpofition to inflammation and abfcefs reigned during the whole of the laft autumn, and ftill prevails.

During the months of October and November abfceffes in the neighbourhood of the bladder and rectum were very common. At prefent the difpofition, in refpect of particular parts, appears to be changed. The extremities feem to be more ftrongly difpofed to inflammatory attacks than the perinæum and contiguous parts. Many have been the inftances that have lately occurred of inflammation, and its confequences, in the arms, legs, fingers, and toes. In fome of them the immediate occafion has been fo very trifling, that it is no wonder it was fuppofed as inadequate to the effect, and that virulence was reforted to in accounting for the confequences. This has been the the cafe, particularly, in thofe little accidents ftudents are liable to in their anatomical refearches. The appearance that is commonly confidered as conclufive in thefe and fimilar cafes, there is reafon to believe ought to be viewed in a very different light. The red ftreaks that are fo alarming to thofe who are ready to admit abforption of poifonous matter, are generally no more than expreffions of extraordinary irritability, the effects of which are propagated in the vafcular parts, along the fides of the abforbents: for they more frequently arife from hurts, in which no poffible virulence can be introduced, than in cafes of inoculation, fyphilis, &c.*

The inftances of inflammation in the perinæum were attended with circumftances that feem interefting, as leading to ufeful diftinctions.

1. Old urethral obftruction was one of the diftinguifhing circumftances. The irrita-

* Numerous facts, fimilar to the following, might be adduced in illuftration of this remark:—A gentleman received a flight hurt in one of his toes; the confequences were, much pain, red ftreaks from the foot along the leg and thigh, and a fmart fever. Means for allaying irritation removed the fymptoms.

tion, ſo common in this caſe, produced the moſt dreadful conſequences during the period mentioned. The inflammation was ſure to proceed to abſceſs, and ſloughing of the cellular ſuſtance and membranous part of the urethra; ſo that in caſe a diſcharge of the matter did not happen, either by nature or art, before the burſting of the urethra, the urine inſinuated itſelf into the ſurrounding parts, and produced extenſive gangrenous deſtruction.

2. When no obſtruction in the urethra had pre-exiſted, on the membranous part becoming compreſſed either by the ſwelling in the perinæum or the preſence of matter, a difficulty in voiding the urine or total ſuppreſſion was the conſequence. On the abatement of the inflammation, or the diſcharge of the matter, the excretion became as free as before.

3. In ſome caſes, from the want of an external opening, the evolved air, and volatile pungent particles from the putrefied cellular membrane, penetrated very extenſively from the perinæum upwards to the pubis and abdomen, exciting inflammation, a gangrenous tendency, and emphyſema with crepitation to the touch.

In

In one of the cafes that occurred I was miftaken in opinion as to the caufe of the fwelling, &c., about the hypogaftric region, in thinking that the urine had efcaped into the cellular membrane. Upon making an ample opening in the perinæum the urine was voided without any trouble, not a drop efcaping by the wound, and the nature of the fymptoms was clearly afcertained*.

Many advantages may be derived from a due confideration of epidemical difpofition.

The nature of fymptoms that may fupervene in wounds, and other accidents, will be better underftood.

The reafons why certain operations at one time prove generally fuccefsful, and at another time otherwife, will appear.

The periods when particular parts of the body

* On confidering thefe complaints every one muft be ftruck with the care and delicacy neceffary in the introduction of the catheter. In a cafe of the fecond defcription, the moment after an ineffectual bold pufh of the inftrument, by a young and inexperienced furgeon, the urine got into the cellular membrane, and produced mortification fo rapidly and to fuch an extent as to terminate fatally, notwithftanding a large opening in the perinæum, and the aid of bark, wine, and opium.

may more or lefs fafely bear operations will be pointed out.

The obfervation fo frequently made at hofpitals, "That many cafes of the fame nature "happen together," will be explained.

Why ulcers, &c., throughout an hofpital, fuffer a change of condition from favourable to unfavourable, and *vice verfa*, at nearly the fame inftant of time, will not appear fo extraordinary*.

The various effects, at different times, of applications, &c., will be lefs often afcribed to wrong caufes.

The power of epidemical caufes may undoubted derive much additional force, or be diminifhed, by many circumftances. The effects of the various degrees and modes of congregation of men in cities and families, and particularly in hofpitals, fhould be attentively confidered, as being highly interefting, and much requiring elucidation †.

* On a fudden fhifting of the wind, for inftance, to the eaft from the oppofite point, I have feen almoft every fore in the hofpital prefently affume a bad afpect.

† Accurate analyfes of the air of hofpitals, fhips, camps, marfhy parts of land, &c., made at various times, could not fail of proving curious and ufeful.

Vete-

Veterinary ſcience would certainly be advanced by accurate journals of events illuſtrating epidemical influence on animals.

The ſubject of epidemics appears to be worthy the attention of particular ſocieties in different countries. Other branches of philoſophical reſearch have been greatly improved by bodies of men particularly engaged in their cultivation: and the ſame ſucceſs may reaſonably be expected from like exertions in reſpect of epidemical cauſes and effects.

December 22d, 1791.

